# Supplementary material for: Impact of the Use of a Larger Forearm Artery on the Efficacy and Safety of Transradial and Transulnar Access: A Randomized Trial with Preprocedural Ultrasonography
Source: J Clin Med. 2020 Nov 9;9(11):3607. doi: 10.3390/jcm9113607 (PMC7697068; doi:10.3390/jcm9113607)
Supplement: Supplementary file 1 [file jcm-09-03607-s001.pdf]

Journal of
Clinical Medicine

MDPI

Supplementary Material

Table S1. Number (%) of patients with secondary endpoints and complications after CAG/PCI at 24-hour and 30-day follow-up. Intention-to-treat analysis.

24 h Follow-Up	Larger UA/RA (*n* = 100)	Smaller UA/RA (*n* = 100)	*p*-Value
RAO/UAO, *n* (%) †	0	10 (10)	0.001 **
Hematoma (grade 4 in EASY scale), *n* (%)	4 (4)	3 (3)	1.0
Stroke/TIA, *n* (%)	0	1 (1)	0.2 **
Major bleeding, *n* (%)	0	0	
IPA, *n* (%)	0	4 (4)	0.121 **
a-v fistula, *n* (%)	3 (3)	1 (1)	0.621 **
Significant stenosis of used artery, *n* (%)	3 (3)	10 (10)	0.044 **
**Intra-arterial complications**			
Perforation of artery in angiography, *n*(%)	10 (9)	10 (12)	0.47
**30-day follow-up**			
RAO/UAO, *n* (%) †	0	15 (15)	0.001 **
Hematoma (grade 4 in EASY scale), *n* (%)	3 (3)	1 (1)	0.6*
Stroke/TIA, *n* (%)	0	0	
Major bleeding, *n* (%)	0	0	
IPA, *n* (%)	0	0	
a-v fistula, *n* (%)	1	0	0.9
Significant stenosis of used artery, *n* (%)	6	13 (13)	0.091 *

a-v fistula—arteriovenous fistula, CAG—coronary angiography, IPA—iatrogenic pseudoaneurysms, PCI—percutaneous coronary intervention, RA—radial artery, RAO—radial artery occlusion, UA—ulnar artery, UAO—ulnar artery occlusion, TIA—transient ischemic attack, †—secondary safety endpoint, *—chi² test, **—Fisher exact test.

Journal of
Clinical Medicine

MDPI

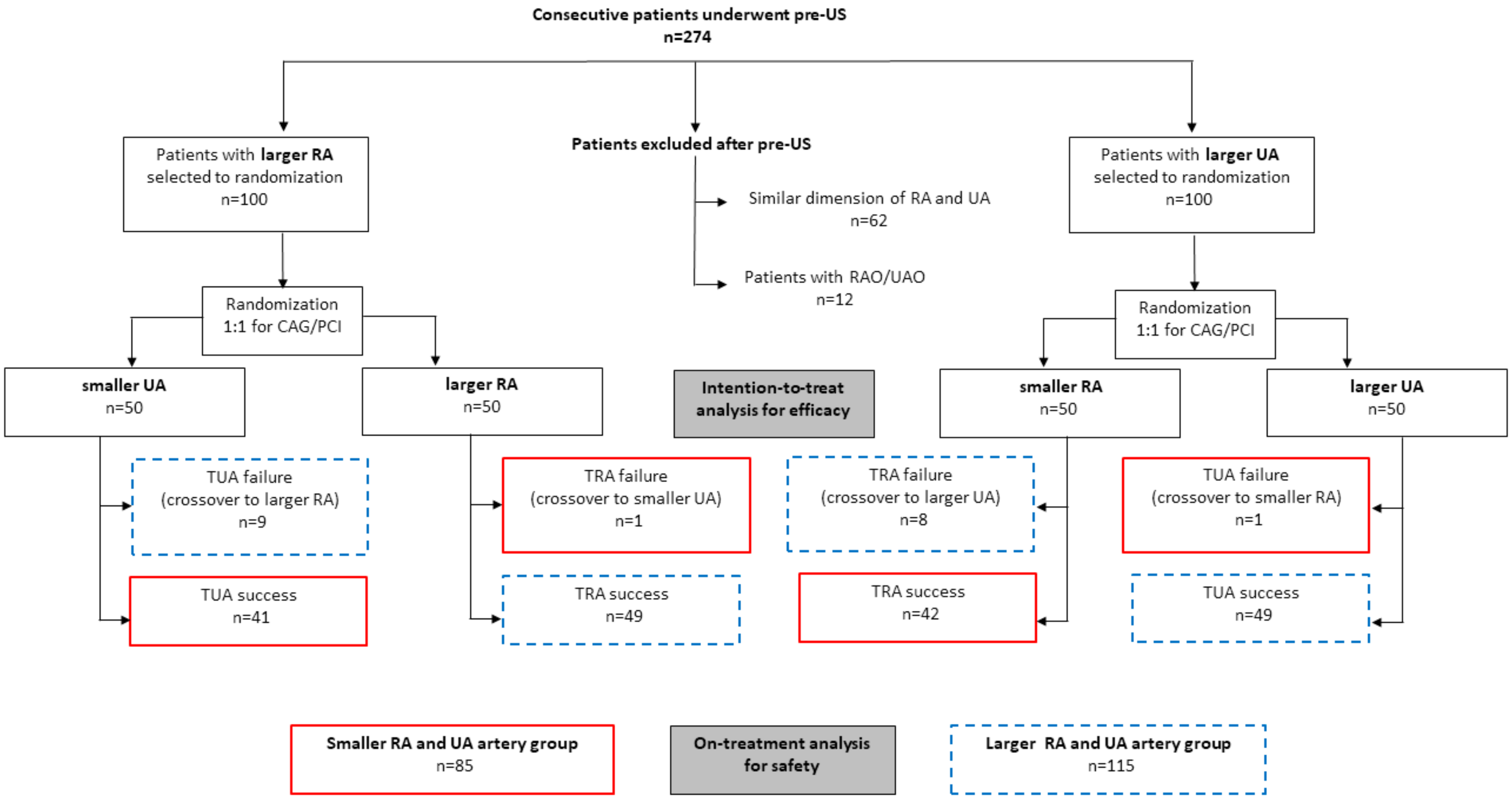

Figure S1. Allocation of patients after layered randomization into larger and smaller artery group.

CAG—coronary angiography, PCI—percutaneous coronary intervention, pre-US—preprocedural ultrasonography, RA—radial artery, RAO—radial artery occlusion, TRA—transradial access, TUA—transulnar access, UA—ulnar artery, UAO—ulnar artery occlusion
